# Leveraging Natural Language Processing to Evaluate Young Adults’ User Experiences with a Digital Sleep Intervention for Alcohol Use

**DOI:** 10.21203/rs.3.rs-3977182/v1

**Published:** 2024-03-28

**Authors:** Frances Griffith, Garrett Ash, Madilyn Augustine, Leah Latimer, Naomi Verne, Nancy Redeker, Stephanie O’Malley, Kelly DeMartini, Lisa Fucito

**Affiliations:** Yale School of Medicine; Yale; Yale School of Medicine; Yale School of Medicine; Yale School of Public Health; University of Connecticut School of Nursing; Yale School of Medicine; Yale School of Medicine; Yale School of Medicine

**Keywords:** natural language processing, digital health, sleep, alcohol use, user experience

## Abstract

Evaluating user experiences with digital interventions is critical to increase uptake and adherence, but traditional methods have limitations. We incorporated natural language processing (NLP) with convergent mixed methods to evaluate a personalized feedback and coaching digital sleep intervention for alcohol risk reduction: ‘Call it a Night’ (*CIAN; N* = 120). In this randomized clinical trial with young adults with heavy drinking, control conditions were *A* + *SM*: web-based advice + active and passive monitoring; and *A*: advice + passive monitoring. Findings converged to show that the *CIAN* treatment condition group found feedback and coaching most helpful, whereas participants across conditions generally found advice helpful. Further, most participants across groups were interested in varied whole-health sleep-related factors besides alcohol use (e.g., physical activity), and many appreciated increased awareness through monitoring with digital tools. All groups had high adherence, satisfaction, and reported feasibility, but participants in *CIAN* and *A* + *SM* reported significantly higher effectiveness than those in *A*. NLP corroborated positive sentiments across groups and added critical insight that sleep, not alcohol use, was a main participant motivator. Digital sleep interventions are an acceptable, novel alcohol treatment strategy, and improving sleep and overall wellness may be important motivations for young adults. Further, NLP provides an efficient convergent method for evaluating experiences with digital interventions.

## Introduction

Most mental health disorders have a peak age of onset during or before young adulthood (18–25 years)^1^. However, less than half of young adults with any mental health disorder receive treatment, with the proportion dropping to around one-third among young adults of color^2^. Clinical services are often unavailable or unaffordable for young adults, and the gap between treatment demand and availability has increased since the COVID-19-pandemic^3^. Further, young adults may not seek treatment for some highly prevalent but normalized mental health concerns, like alcohol use disorders (AUDs) and heavy (“binge”) drinking^2,4–5^. In contrast, young adults show concern for other common wellness issues, like sleep quality, that are related to alcohol use^6–7^. In a sample of young adults with similar levels of sleep complaints and heavy drinking, most (80%) were concerned about sleep whereas very few (5%) were concerned about drinking^8^. Therefore, sleep interventions could offer a more appealing alternative for young adults facing sensitive issues like alcohol risk^9^.

Digital interventions can also increase appeal, access, and convenience of mental health treatment for young adults^10^. This population makes extensive use of digital health tools compared to other adults^11^, with generally positive experiences^12^. Despite a saturated market of mobile mental health apps and other digital tools, user ratings may be discrepant and uninformative to app designers and potential users alike^13^. Over 95% of users stop using most mobile mental health apps after 30 days^14^ and adherence and engagement are generally low^15^. In the case of sleep and alcohol apps, user engagement may increase with features like personalized feedback, interaction and support from providers, self-monitoring, and user-friendliness^16–19^. Further, gamification may significantly reduce attrition in mobile mental health app use in general^20^. With the incorporation of machine learning, apps are also becoming increasingly more personalized. Assessing both users’ perspectives and intervention quality is critical to promote higher treatment uptake and adherence in a rapidly growing, global digital health market^21^. Therefore, implementing precise, consistent evaluations of user experiences represents a significant gap in digital intervention research.

Incorporating natural language processing (NLP) with convergent mixed methods can support user-centered design for digital health interventions^22^. Natural language processing is an umbrella term for a growing suite of machine learning methods that uses artificial intelligence (AI) to understand (e.g., summarize, retrieve information) and/or generate language content. Traditional evaluation methods, such as qualitative thematic analysis of exit interviews, can reveal nuance and detail, but can be impractical, especially with large sample sizes^23^. Further, exit surveys using Likert-type satisfaction scales may be subject to response bias. A hybrid approach combining NLP, quantitative survey analysis, and targeted qualitative interview analysis, may reveal broad and rich user experiences^23^ while maximizing researcher time and effort.

**Figure 1:**
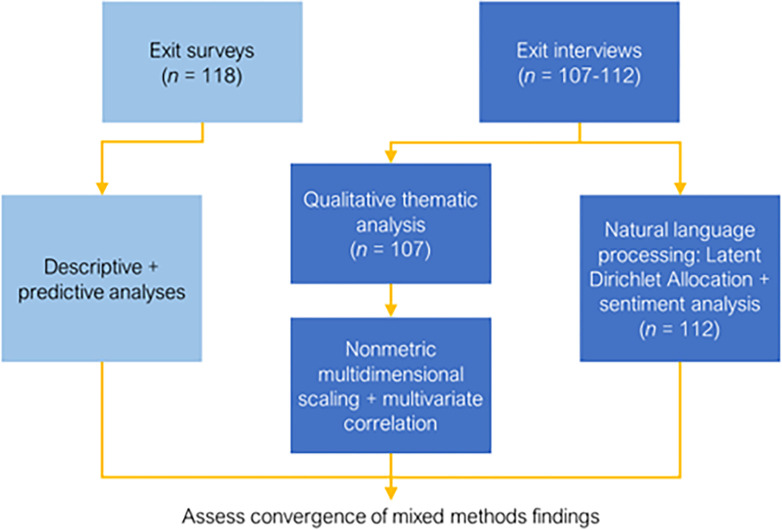
Hybrid convergent mixed methods with NLP for user experience evaluation. Descriptive and predictive analyses were used with exit surveys. Targeted qualitative thematic analysis, post hoc NMDS and multivariate correlation, and NLP were used with exit interviews.

The current study used convergent mixed methods to evaluate user experiences in a randomized clinical trial of “Call it a Night” (*CIAN*) and two control conditions (see Fig. 1). *CIAN* is a personalized feedback and coaching digital sleep intervention for young adults that addresses heavy alcohol and other substance use (NCT #03658954). In the parent trial^6^, *CIAN* was tested against control conditions comprising either web-based advice only (*A*) or advice + active diary self-monitoring (*A* + *SM*). All participants wore sleep and alcohol biosensors. NLP methods, particularly topic modeling analysis (Latent Dirichlet Allocation) and sentiment analysis, were selected to assess convergence with qualitative thematic analysis of exit interviews and descriptive and predictive analysis with exit surveys. The aims of the study were to leverage these NLP evaluation methods to:

1. characterize young adults’ user experiences during the digital sleep interventions for heavy drinking and
2. determine whether user experiences varied with their demography (e.g., age, race, gender, student status, psychiatric diagnoses) or trial condition.

## Results

### Sample

We recruited 120 trial participants, and 118 completed the exit survey. Half (51%) were female and 80% were white with a mean age of 21.14 years (see Table 1). Three-fourths (74%) were college students. Most (81%) met lifetime criteria for any MHD or substance use disorder (SUD) based on a diagnostic interview, and most also met lifetime criteria for an AUD (72%).

### Thematic Analysis of Exit Interviews

Qualitative thematic analysis of exit interviews resulted in overarching thematic categories related to helpfulness of different intervention components and suggestions from participants (see Table 2 for themes and salience; see Supplemental Materials for all thematic analysis definitions and exemplar quotes).

### Web-based Sleep Advice (Website): Helpful and Unhelpful Aspects (n = 107)

Participants highlighted various aspects of the web-based advice intervention, both helpful and unhelpful. The most mentioned helpful aspects were *General helpfulness of the website and Helpful sleep-related information*: “I think the particularly helpful ones [tips] were…things to do when you wake up, because I think …when I wake up, I just don’t want to get out of bed, so I’ll…stay in bed on my phone for a long time.” Participants appreciated learning *New information from the website, Reminders of known information, and Usefulness of website strategies*: “It [the website] talked about…your body temperature… keeping a window open or having a fan to keep your body [cool].” Others noted helpful aspects of *Website understandability, User-friendliness of website, Helpful alcohol-related information, and Memorability of information*. By contrast, relatively few participants described unhelpful aspects. Some mentioned *Lack of new information* as a drawback, while others described *Irrelevance of information*, such as content related to cannabis and sleep. A few participants reported *Difficulty implementing strategies* recommended on the website.

### Passive Monitoring (Biosensors): Helpful, Neutral, and Unhelpful Aspects (n = 107)

Participants across trial groups provided comments on whether wearing the biosensors (transdermal alcohol ankle monitor and sleep watch) was helpful on its own without consideration of the data collected. Most described *Neutral aspects of biosensors* and did not find them helpful or unhelpful without data. Among those who found the biosensors helpful on their own, the most mentioned aspect was that the *Ankle biosensor increased awareness* of their alcohol use: “The physical monitors all over you…they definitely make you…think like, ‘Wow…should I really be drinking this much?’” Likewise, others noted the *General helpfulness of the biosensor(s)* or that the *Watch biosensor increased awareness of* their sleep patterns irrespective of data obtained: “If I saw my sleep watch before I was going to bed, I was like, ‘You know what, I should probably try and go to bed early tonight.’” Among those who found one or both biosensors unhelpful, the most common complaint was *Burdensomeness of ankle biosensor*, including itchiness, discomfort, and difficulty completing some activities: “I just really didn’t like the [ankle] device…couldn’t run as much.” Other unhelpful aspects mentioned were *Unhelpful without data feedback, Lack of behavior change, Burdensomeness of watch biosensor* (e.g., forgetting to push buttons), Stigma of ankle biosensor (e.g., connection to court-ordered monitors), and *Ankle biosensor increased drinking*.

### Active Self-Monitoring (Diaries): Helpful, Unhelpful, and Neutral Aspects (n = 78)

CIAN and *A* + *SM* participants commented on the helpfulness of completing diaries, a component of their study conditions. Most described one or more helpful aspects, most commonly that diaries *Increased mindfulness of sleep*: “I found it…helpful to…realize it [my sleep schedule] and…just reflect on…my lifestyle and…how much sleep I’m actually getting.” Others noted the *General helpfulness of diaries* and *Increased mindfulness of alcohol use*: “I…never really…used to like keeping track [of drinking]. So, I’d… drink a lot, and then I’d feel too drunk.” Some noted the helpfulness of the *New experience of keeping a diary, the Ease of answering questions* in the diaries, or the *Motivation to change behaviors* prompted by diaries. Fewer participants noted unhelpful aspects of diaries, such as *Challenges answering questions* in the diaries, *Lack of behavior change*, and *Lack of new information* (i.e., self-monitoring did not increase awareness of patterns nor motivate them to change behaviors. Some also noted a *Neutral aspect of diaries*, they were neither helpful nor unhelpful.

### Personalized, Tailored Coaching (Feedback): Helpful and Unhelpful Aspects (n = 50)

Participants in the *CIAN* condition with personalized feedback and coaching largely described helpful aspects of the feedback and coaching intervention component. Almost all *CIAN* group participants described the *Helpfulness of personalization*, the individualized nature of reports and suggestions based on monitoring data: “Having that sort of feedback, both the data and just the explanation behind it, I think that’s a really…unique insight into…a part of yourself that…most people wouldn’t ever really get to actually see mapped out.” Most also noted aspects, such as the *General helpfulness of feedback* and *Helpful data presentations*: “She [the coach] made it very easy for me to understand the charts and what everything meant.” Only a few described one unhelpful aspect of feedback: *Lack of new information*, i.e., reports did not contribute new insights into their sleep or drinking.

### Most Influential Interventions

Each participant was asked to select which received intervention component(s) were most influential for behavior change. In order from most to least helpful, participants selected personalized feedback/tailored coaching (i.e., three-fourths of *CIAN* participants), the website (i.e., over one-third of all participants), diaries (i.e., almost one-third of *CIAN* and *A* + *SM* participants), and biosensors (i.e., less than one-fifth of all participants).

### Suggestions for Future Interventions

During exit interviews, participants also offered suggestions for current and future intervention improvement. Many participants were interested in measuring additional sleep-related factors, most commonly that they *Want diet and exercise-related feedback*: “[I’d like to know] what time when you exercise, how it affects your sleep…what that does to…your whole body chemistry, everything.” Others mentioned they *Want caffeine-related feedback*, *Want environment-related feedback* (e.g., impacts of light, noise, and temperature on sleep), or *Want other potential feedback* (e.g., cognitive alertness, tobacco, and stress): “Maybe you can see how stressed you are during… the week. Just get those numbers, and then I think that would be pretty interesting to figure [out].” To receive additional feedback, most responded that they would be *Willing to wear more devices* and *Willing to complete new diary questions*. Fewer stated they were *Unwilling to add a question and/or device*, usually disinterest in another ankle biosensor. Several volunteered suggestions for received interventions in the current study. These included *Improvements for feedback* (e.g., combined, summary report for both intervention weeks, *Improvements for diaries* (e.g., higher pay per diary)*, Improvements for biosensors* (e.g., eliminating ankle biosensors, measuring GPS location), and *Improvements for website* (e.g., wider distribution, alternative summaries).

### Nonmetric Multidimensional Scaling of Interview Themes

**Figure 2:**
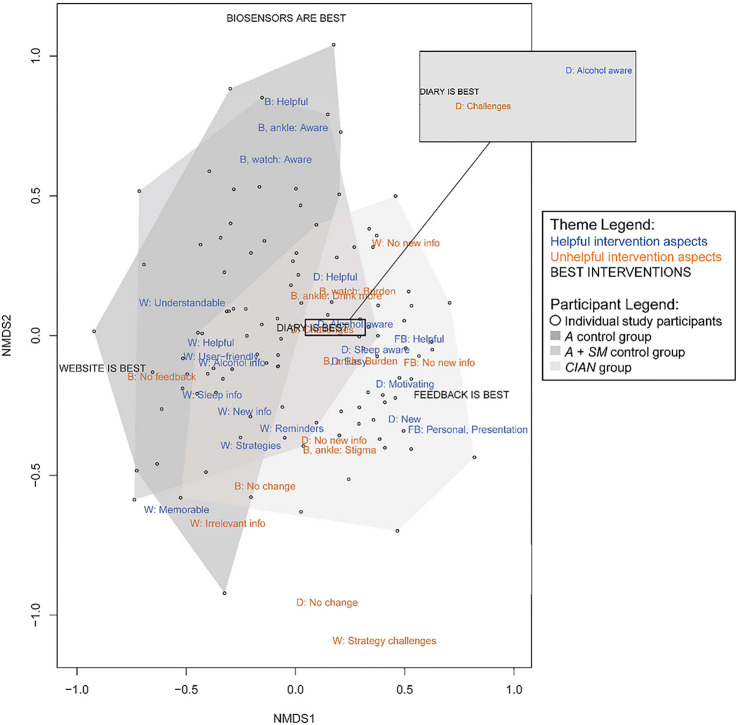
NMDS ordination plot of user experience themes from exit interviews. This ordination plot shows study participants (points) and themes (text) from thematic analysis mapped on the first two dimensions of the NMDS ordination. Polygons drawn around participant points indicate trial groups. Themes about helpful aspects of interventions are shown in blue, themes about unhelpful aspects of interventions are shown in orange, and themes about self-reported most influential interventions are shown in black. “W” stands for website, “B” stands for biosensors, “D” stands for diary, and “FB” stands for feedback.

**Figure 3:**
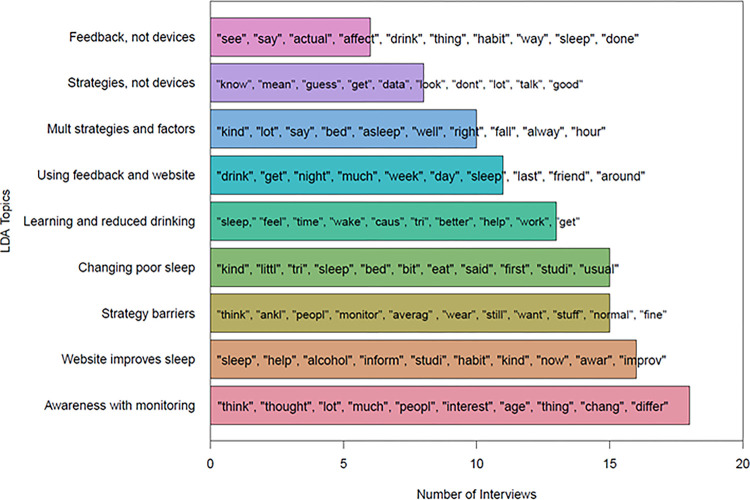
LDA topic and term frequency in exit interviews. This bar plot shows the frequency of the topics from the LDA among 112 exit interviews and feedback transcripts. The ten most common terms within each topic are shown in the order of frequency.

We used multivariate analysis, nonmetric multidimensional scaling (NMDS)^24^, to visualize the interrelationships among qualitative themes and participants. NMDS is an ordination method that condenses variation in matrices, such as participant-by-theme frequency, to a small number of orthogonal dimensions^25^. We used five orthogonal dimensions based on stress-level testing, and the first two dimensions are plotted in vector space in Fig. 2. Also, multivariate correlational analysis allowed us to fit participant characteristics as factors to the NMDS ordination of themes. Trial group (*R*^2^ = .29, *p* = .001) and lifetime history of any MHD/SUD (*R*^2^ = .03, *p* = .04) were significantly correlated with NMDS scores. That is, participants’ statements about study interventions in their exit interviews were associated with trial group and diagnostic history. Other participant characteristics (e.g., age, gender, race, ethnicity, student status) were not associated with NMDS scores. In the NMDS shown in Fig. 3, *CIAN* and *A* + *SM* participants were closer to themes of feedback and diary helpfulness than website or biosensor helpfulness in vector space, which suggests their preference for feedback and diaries. Conversely, control *A* and *A* + *SM* participants were closer to themes of helpful website aspects in vector space than *CIAN* participants, and control participants were also significantly more likely to state these themes during interviews (*X*^*2*^ = 27.34, *p* < .001).

### Natural Language Processing of Exit Interviews (n = 112)

We also used quantitative Latent Dirichlet Allocation (LDA), a topic modeling analysis that identifies the likelihood of terms occurring in topics and topics occurring in documents^26^, with an expanded dataset of 112 participants’ exit interviews and coaching transcripts and identified nine topics across participants’ statements (see Fig. 3; see Supplemental Material for all topic definitions and example quotes). The goals of NLP analyses were to help qualitative thematic analysis be more targeted and assess convergence of findings with thematic analysis and exit survey analyses. These topics did not vary significantly by trial condition (*X*^*2*^ = 16.72, *p* = .40). We found that *Awareness with monitoring* was the most likely topic in the largest number of interviews (*n* = 18, 16%). This included participants’ perceptions that both active diary self-monitoring and passive biosensor monitoring increased their awareness and mindfulness of their behaviors. As one participant stated, “Knowing that I had to do the sleep diary the next day and…people were watching what I was doing…I was just more aware of my habits.”

Other common topics focused on web-based advice, especially sleep strategies. The topic, *Website improves sleep* (*n* = 16, 14%), included experiences of beginning to improve sleep using sleep strategies, such as consistent sleep schedules. A participant described, “My sleeping habits were definitely awful before now, and I think they’ve improved a lot, since starting this [study]…I’m starting to go to bed earlier and…reduce my activities before bed.” Almost as many interviews were most likely to include the topic, *Strategy barriers* (*n* = 15, 13%), or descriptions of challenges implementing sleep strategies, such as situational factors (e.g., work and school schedules, dormitory environment) or personal factors (e.g., memory, motivation). The topic *Changing poor sleep* (*n* = 15, 13%), included descriptions of poor sleep quality or nonrestorative sleep, how this motivated study participation, and a desire to learn about different sleep factors.

Other topics focused on participants’ interest in gaining more wellness-related knowledge to change sleep or alcohol use. Thirteen interviews (12%) were most likely to include the topic *Learning and reduced drinking*, which focused on gaining new information from digital interventions and reducing drinking given its impacts on sleep. The topic *Using feedback and website* (*n* = 11, 10%) included participants’ accounts of how they integrated personalized feedback and coaching with web-based advice, including information and strategies, to alter their sleep and alcohol use. The topic *Multiple strategies and factors* (*n* = 10, 9%) focused on participants’ curiosity and interest in the impacts of varied sleep-related factors, such as situations, environments, substances other than alcohol, and diet and exercise.

The two least frequent topics included the burden of wearable biosensor devices. The topic *Strategies, not devices* (*n* = 8, 7%) focused on helpful website aspects, attempts to implement sleep strategies, and challenges with wearable devices, especially the transdermal ankle monitor. Similarly, the topic *Feedback, not devices* (*n* = 6, 5%) focused on the benefits of personalized feedback and coaching while also discussing difficulties with wearable devices.

**Figure 4:**
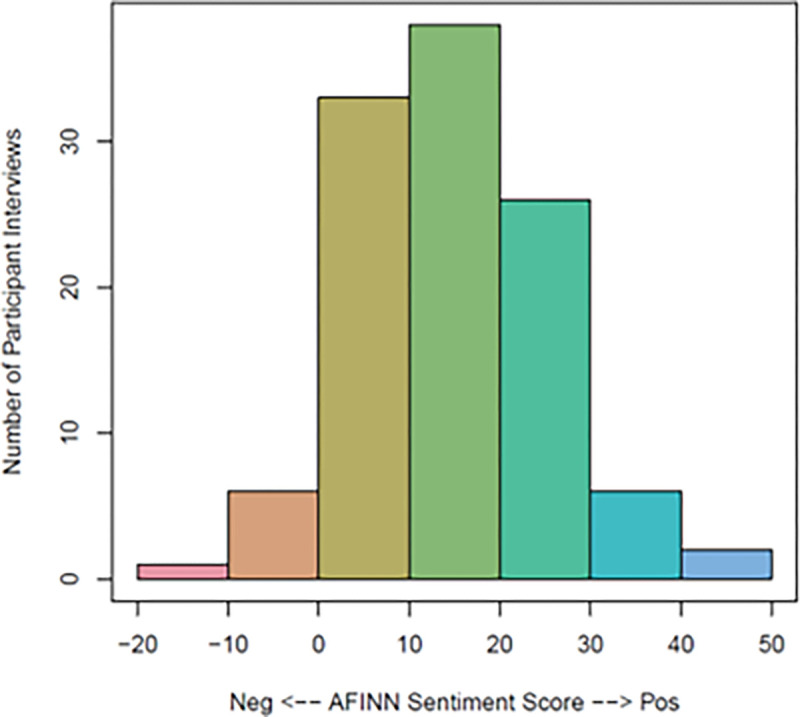
Exit interview sentiment frequency. This histogram shows the frequency of interviews’ net sentiment scores based on the AFINN lexicon^42^ among 112 exit interviews and feedback transcripts.

The sentiment scores of exit interviews and transcripts (*n* = 112) were generally positive (*M* = 15.07, *SD* = 10.54) and ranged from − 16 to 47 (see Fig. 4). Positive vs. negative sentiment scores reflect the aggregate valence of participants’ word usage in exit interviews, so “0” reflects a neutral net score in an interview. Sentiment scores did not vary as a function of trial condition (*CIAN, A* + *SM*, and *A*; *F*(2, 109) = 0.78, *p* = .46). Positive sentiment scores also did not vary by demographic characteristics, including gender (*F*(1, 110) = 0.62, *p* = .43), age (*b* = 0.03, *t* = 0.06, *p* = .95), race (*F*(4, 107) = 1.23, *p* = .30), ethnicity (*F*(1, 110) = 0.20, *p* = .66), student status (*F*(3, 108) = 0.75, *p* = .53), and history of any MHD/SUD (*F*(1, 110) = 0.23, *p* = .63). However, the word length of each document was positively correlated with sentiment scores (*r* = 0.21, *p* = .03), indicating a tendency for participants who spoke at longer length to speak more positively about their experiences. To boost rigor and account for this correlation, we derived a valence-per-word score for each document and reran sentiment comparisons across condition and demographic groups with the valence-per-word score as the outcome. There were still no between-group differences (*p* > 0.05).

### Exit Survey (n = 118)

On the exit survey, participants across conditions (*CIAN, A* + *SM*, and *A*) reported generally positive user experiences (*n* = 118; see Table 3). On a 5-point scale, participants reported high overall program satisfaction, website helpfulness, diary helpfulness, and feedback helpfulness. Mean feasibility ratings were generally high for the overall program, website, watch biosensor, and diaries. However, mean feasibility ratings for the ankle biosensor were lower based on visual inspection, especially due to interference with clothing, feeling uncomfortable, and being noticeable. Perceived effectiveness was higher among *CIAN* (Δ = 0.48, *p* = .008) and *A* + *SM* participants (Δ = 0.55, *p* < .001) than A participants (*F*(2, 115) = 8.45, *p* < .001). Further, young adults with a lifetime history of any MHD/SUD rated their intervention as more effective on average than those without diagnoses (*F*(1, 116) = 4.64, *p* < .001; Δ = 0.32, *p* = .03).

Adherence to interventions was also high across study phases. Almost all participants (98%) completed the two-week intervention phase, and 96% completed the 12-week follow-up appointment. Regarding monitoring activities, 98% of participants wore the sleep watch biosensor for 14 days, and 95% wore the alcohol ankle biosensor for 14 days. Further, 95% of participants in the *CIAN* and *A* + *SM* groups completed their assigned 14 days of active diary self-monitoring of sleep and alcohol use.

## Discussion

The current study used an innovative hybrid approach in which NLP findings converged with and corroborated more traditional mixed methods to efficiently evaluate user experiences with a digital sleep intervention to reduce drinking. Major findings showed that participants across conditions generally found all intervention components helpful, satisfying, and feasible, but the *CIAN* group markedly preferred the personalized feedback and tailored coaching with a health professional they received to other components. Convergent results also underscored young adults’ strong interest in gaining a more holistic picture of their wellness in the future and increasing their awareness using monitoring through diaries and biosensors. Our hybrid approach using NLP enabled qualitative thematic analysis to target specific interview questions more efficiently, optimizing researcher’s time. Further, assessing the convergence of findings between NLP and other methods helped address potential researcher bias in thematic analysis and potential user response bias in exit surveys.

### The Importance of Personalized and Interactive Feedback and Coaching

Mixed methods findings on user experiences, including NLP, emphasized greater effectiveness and satisfaction with personalized feedback and coaching than other intervention components despite generally favorable experiences with web-based advice, active diary self-monitoring, and passive biosensor monitoring. *CIAN* and *A* + *SM* group participants who received feedback and/or actively self-monitored (diaries) rated their intervention as more effective than *A* control participants in the exit survey. Topic modeling (LDA) and thematic analysis showed that some participants found web-based advice strategies difficult to implement, and negative themes about web-based advice were associated more with *CIAN* participants.

Our study adds insight to extant literature on young adults’ preferences for interactive digital interventions, including alcohol and sleep treatment. An umbrella overview of meta-analyses and systematic reviews^21^ found that digital interventions offering interaction with a health professional or other social support had higher adherence and effectiveness and lower attrition. Mobile alcohol treatment apps providing personalized feedback may be especially engaging^17^. Further, recent research on mobile sleep apps^16, 18–19^ highlights increased engagement and satisfaction with personalized feedback and communication with health professionals. Our study adds findings that individually tailored coaching from a health professional significantly enhanced participants’ experience interpreting digital health data, which likely supported their understanding. Current research also shows that user-friendliness^17^ and evidence-based information^18^ increase engagement, which was consistent with our finding that web-based advice was considered helpful and user-friendly, especially by control groups. Therefore, whereas web-based advice and other automated interventions are likely to be helpful to many young adults, tailored feedback and coaching with individualized data are likely to be preferable in the current digital health market.

### Empowering Young Adults with Digital Health Tools for General Wellness

Consistent with prior findings^11^, our results showed that young adults particularly want to use digital tools to pursue holistic health and wellness goals. Thematic analysis and LDA with interviews converged to show that participants are interested in understanding varied other lifestyle factors that impact sleep beyond alcohol (e.g., caffeine, diet, exercise, stress, environment). Most participants expressed a willingness to use additional digital tools (e.g., more biosensors, additional diary self-monitoring) to attain whole-health, personalized feedback. LDA results highlighted greater perceived awareness and mindfulness of sleep and drinking through diary self-monitoring and/or wearing biosensors. Thus, while young adults are interested in and capable of using varied digital health tools, incorporating appealing treatment foci could be important to increase young adults’ generally low engagement and adherence^15^.

Our study suggests that digital interventions improving overall health may represent a more appealing treatment approach to young adults for potentially stigmatizing diagnoses, such as AUDs. Most available digital SUD interventions for young adults involve web-based advice explicitly targeting alcohol use to the exclusion of other substance use or wellness factors^27^. Whereas young adults may not be concerned about alcohol use or seek traditional alcohol treatment^5^, they are concerned about improving sleep quality^6,8^ and other aspects of their general wellness. In this study, LDA with exit interviews showed that participants commonly joined the study to improve their sleep quality rather than address their alcohol use. Further, survey and interview thematic analysis results, respectively, showed that participants with a lifetime history of an MHD/SUD found the current intervention to be especially effective and described intervention helpfulness differently than those with no diagnoses. Therefore, digital interventions, like the current study which focused more explicitly on improving wellness (e.g., enhancing sleep quality), may be especially engaging to young adults with AUDs or other diagnoses who have not presented to treatment.

### Implications and Recommendations

Our study has implications for clinical researchers and mobile app designers making user-centered app designs. User experience evaluations of digital health tools should incorporate a hybrid, convergent approach to optimize depth, breadth, and efficiency. Each user experience evaluation method has distinct strengths and potential weaknesses^23^. Thematic analysis of exit interviews can provide detailed information across complex subjective experiences. In the current study, along with post hoc analyses, thematic analysis revealed more distinctions between perceived helpfulness of different intervention components than any other method. However, as sample sizes increase, qualitative thematic analysis can become impractical and unfeasible. Our use of NLP topic modeling (LDA) to derive overarching topics across interview questions enabled us to target and abbreviate our thematic analysis to interview questions focusing on intervention component helpfulness. Exit surveys are also time-efficient like NLP but may be subject to user response bias. NLP, specifically sentiment analysis, helped confirm the generally positive perceptions derived from exit survey analyses.

Our convergent evaluation findings also have specific implications for digital intervention design. Young adults are enthusiastic about receiving a broad, holistic picture of factors impacting their wellness, including different aspects of their lifestyle, in addition to appreciating individualized and specific coaching. Therefore, mobile mental health apps and linked biosensors should have a variety of choices for active and passive monitoring that can be tailored to user feasibility and interest. Digital tools should integrate seamlessly into young adults’ schedules that often include exercise, which may make ankle transdermal monitors challenging and potentially stigmatizing^28^ compared to watch biosensors. Also, digital interventions, including for sleep and alcohol use, should empower young adults to actively explore their wellness with support from a health professional. While participants in the current study valued the mindfulness derived from digital health monitoring, they preferred to also receive personalized data reports with explanations and suggested health tips from a coach. Some individuals may lack the expertise to interpret their health data or to devise action plans for improvement. Thus, digital interventions should include mechanisms for interactive feedback, such as an on-call health provider, pre-scheduled video or text check-ins with a health provider, or a chatbot that provides a spectrum of suggestions and individual data descriptions.

Digital interventions for specific, sensitive clinical issues, like heavy drinking, could engage more young adults via a focus on related general health and wellness behaviors, like sleep. Interventions that increase accessibility and engagement for young adults are important given that only half of young adults with psychiatric disorders currently receive any mental health treatment^2^. Digital sleep interventions for reduced drinking can target two risky, prevalent, and modifiable issues: drinking and sleep problems. Young adults commonly experience AUDs, binge drinking^2^, and sleep problems^7^. Further, sleep problems in adolescence lead to heavier drinking and AUDs in adulthood^29–31^. Notably, young adults who drink heavily may be less concerned about their drinking than their sleep^8^. In general, digital tools that explicitly focus on less sensitive behavioral goals, like improved sleep, with implications for improving potentially stigmatized goals, like reduced drinking, are likely to be more appealing and engaging to young adults.

### Strengths and Limitations

Distinct strengths of the current study included a relatively large sample size, especially for a qualitative analysis of exit interviews; generally high adherence across intervention phases and groups; and minor amounts of missing data. Each of these attributes increased the validity of findings. Further, our hybrid, convergent approach incorporating NLP represents an important strength as these methods are promising and relatively new in digital medicine^22–23^. Our research also had notable limitations. Although missing data were minor, it is possible that additional themes would have emerged in a complete set of exit interviews. NLP helped address response bias in exit survey items, but survey responses were not normally distributed and generally skewed towards higher responses. Our sample was primarily college students, so it may not be representative of young adults in general. Further, study participants were paid, so implementation and uptake may differ in clinical or other naturalistic settings.

## Conclusion

The current evaluation demonstrates the value of NLP in convergent mixed methods to efficiently capture broad and nuanced user experiences with digital health interventions. Specifically, our results show that digital sleep interventions for heavy drinking may increase appeal and access to alcohol treatment for young adults, especially when they include tailored coaching. Broadly, the current findings emphasize the importance of digital tools for young adults that provide a holistic, dynamic view of health coupled with interactive, individualized feedback. Consistent and precise evaluations that leverage user feedback are critical to support higher uptake and adherence to effective digital health interventions, which can begin to address the mental health treatment demand gap for young adults.

## Methods

### Study design

In the current study, we evaluated user experiences during a randomized clinical trial of *CIAN*, a novel, two-session personalized feedback and coaching digital sleep intervention developed for young adults to reduce drinking (*N* = 120). Participants were randomly assigned to one of three conditions using a 2:1:1 ratio for two weeks: 1) *CIAN*: two-session “Call it a Night” intervention (*n* = 60) or 2) one of two control conditions, *A*: brief advice only (*n* = 30) or *A* + *SM*: brief advice + active sleep diary self-monitoring (*n* = 30). For this outpatient study in participants’ natural environments, all wore sleep and alcohol biosensors daily for two weeks and viewed a two-module web-based sleep advice program during brief, midweek research appointments. There were no significant differences between the trial groups on any demographic variable (i.e., age, gender, race, ethnicity, student status, lifetime history of psychiatric diagnosis, or baseline alcohol use). Of the participants, 118 completed the exit survey and 107 completed the exit interview with study staff.

To evaluate user experiences, we used convergent mixed methods with NLP following the two-week intervention phase. Exit surveys and interviews were conducted in parallel with participants across trial conditions to attain both broad and deep perspectives on satisfaction and intervention acceptability and feasibility. Both qualitative thematic analysis and quantitative NLP examined exit interviews. Further, in line with convergent mixed methodology, exit survey results were analyzed using descriptive and predictive analyses to compare these findings with exit interview results. This study was approved by the Institutional Review Board of Yale University (IRB# 2000021048). Additional study details can be found in the published study protocol paper^6^.

### Recruitment

To be eligible for the *CIAN* clinical trial, participants needed to a) be 18–25 years of age, b) be fluent in English, c) self-report three or more heavy drinking occasions in the last two weeks (i.e., 5 or more drinks on one occasion for men or 4 or more for women), d) score at risk of harm from drinking on the AUDIT-C^32–33^, and d) self-report sleep concerns. Exclusion criteria are described further in the study protocol paper^6^. Participants were recruited using online (e.g., Facebook, Instagram, Snapchat) and in-person advertisements placed around the local community. Online advertisements and flyers directed individuals to a web-based pre-screening survey, and those who met pre-screening eligibility were invited to an intake visit for final eligibility determination.

### Exit Survey and Interview

Upon completion of the two-week intervention phase, we asked participants to complete a self-report exit survey (see Supplemental Material for exit survey items). The exit survey included Likert-type and yes/no questions to assess user experiences, including overall satisfaction, intervention helpfulness and effectiveness, and intervention feasibility. Participants were also asked to detail areas for improvement in open-ended response items.

Participants across trial groups also completed semi-structured exit interviews to provide information about their subjective user experiences (see Supplemental Material for exit interview protocol). Exit interviews were administered by three research staff members (two men, one woman), who were trained in the protocol and familiar with study interventions. The exit interview protocol included questions about the following topics: participants’ sleep and alcohol use before and after the study, perceptions of these behaviors relative to their peers, use of sleep hygiene strategies from the intervention, helpfulness of intervention components, the impact of payment on study participation, what drew them to the study, and suggestions for intervention improvement or future studies targeting sleep or alcohol use.

### Data Analysis

We conducted both qualitative thematic analysis and quantitative NLP with exit interviews. For thematic analysis, we used Braun and Clarke’s six-step process^34^, during which we used a recursive and iterative team coding process to ensure rigor and trustworthiness in the process of identifying, naming, and categorizing recurrent themes. Two members of the research team completed an initial open coding of the entire text of a large subset of exit interviews (*n* = 80) to derive initial themes. Then, guided by findings from NLP topic modeling analysis, the current thematic analysis targeted key portions of the exit interviews, including questions related to helpfulness of intervention components and participants’ suggestions. Three research team members engaged in a rigorous team coding process of the entire dataset of completed two-week exit interviews (*n* = 107). Using an initial codebook based on previous first-round, open thematic coding, these three researchers independently coded a random selection of 20% of the entire interview dataset (*n* = 21) to evaluate interrater reliability. All themes had percent agreement of 95% or greater, but an extensive reconciliation meeting was still undertaken to audit the initial codebook and resolve any disagreements until an overall kappa exceeding 0.7 was reached between each pair of coders. The remaining exit interviews were divided among the three researchers to code using the revised codebook. Auditing was used throughout the qualitative process to ensure transparency, including field notes maintained by each coder and reviewed by other coders and ongoing check-ins with the entire research team.

To visualize the interrelationship between participants and themes, we conducted NMDS with theme frequency counts using the *vegan* package^24^ in the programming language R^35^. NMDS is a multivariate ordination method that condenses variation in matrices to a small number of orthogonal dimensions^25^. We selected NMDS for post-hoc analysis of themes because it does not place assumptions of normality on frequency count data compared to other multidimensional scaling methods^25^. To assess the goodness of fit when selecting the number of orthogonal dimensions, we used stress level cutoffs and a stress plot (Shepard diagram)^24^. We selected five orthogonal dimensions, resulting in a good to fair stress score of .13, which is likely to result in minimal distortion^36^. As shown in Fig. 2, we used a multivariate correlation analysis with vegan in R to fit factors (trial condition and participant gender, race, ethnicity, student status, and diagnosis history) and a vector (participant age) to the NMDS ordination of participants and themes. This enabled us to assess whether participants’ user experiences (as interview themes) varied significantly with their assigned trial condition or personal characteristics.

For NLP of exit interviews, we conducted topic modeling^26^ and sentiment analyses^37^ with an expanded dataset of all of participants’ statements during completed exit interviews and feedback session transcripts (*n* = 112). LDA with the *topicmodels* package^26^ in R was used for topic modeling analysis. To boost rigor and ensure the most parsimonious number of topics (*k*) were used for LDA, we undertook a preliminary analysis with three model selection methods to test different *k* values^38–40^ in the *ldatuning* package^41^ in R and ultimately selected nine topics. Text in participants’ statements was preprocessed before NLP, including removing punctuation, numbers, capitalization, and English ‘stopwords’ as well as stemming all remaining words. The LDA determined which of the nine topics was most likely to occur in each document and which terms were most likely to occur in each topic. Topics were systematically named by closely reading five or more interview transcripts determined most likely to contain the topic by LDA. Chi-squared tests determined whether topics varied with participants’ assigned trial condition.

For sentiment analysis, we used the AFINN lexicon^42^ and the *syuzhet* package^37^ in R. The AFINN lexicon is a well-established and commonly used sentiment lexicon^37^ that assigns a valence score from − 5 to 5 to selected terms, and documents are scored based on an aggregate of their terms’ scores. An aggregate AFINN sentiment score of “0” for a document indicates neutral term valence. Bivariate regression models, including analysis of variance (ANOVA), were used to assess whether participants’ trial condition or personal characteristics predicted the sentiment of their statements during exit interviews.

Descriptive and predictive analyses were conducted with exit survey results. These included summary statistics of each item and linear modeling to determine if participants’ trial condition or personal characteristics predicted responses to exit survey items.

## Figures and Tables

**Table 1:** CIAN Exit Survey Sample Characteristics (n = 118)

Intervention group	*A*	*A+SM*	*CIAN*	Total ^a^
Sample characteristic	*n* (% of 29)	*n* (% of 29)	*n* (% of 60)	*n* (% of 118)
Gender				
Female	14 (48.3)	16 (55.2)	30 (50.0)	60 (50.8)
Male	15 (51.7)	13 (44.8)	30 (50.0)	58 (49.2)
Race				
Asian	3 (10.3)	2 (6.9)	5 (8.3)	10 (8.5)
Black	1 (3.4)	1 (3.4)	7 (11.7)	9 (7.6)
White	25 (86.2)	24 (82.8)	45 (75.0)	94 (79.7)
Multiracial	0 (0.0)	0 (0.0)	1 (1.7)	1 (0.8)
Other	0 (0.0)	2 (6.9)	2 (3.3)	4 (3.4)
Ethnicity				
Not Hispanic, Latine	24 (82.8)	24 (82.8)	51 (85.0)	99 (83.9)
Hispanic, Latine	5 (17.2)	5 (17.2)	9 (15.0)	19 (16.1)
Student status				
Student	21 (72.4)	23 (79.3)	43 (71.7)	87 (73.7)
Non-student	8 (27.6)	6 (20.7)	17 (28.3)	31 (26.3)
History of mental health or substance use diagnosis				
Yes	23 (79.3)	25 (86.2)	48 (80.0)	96 (81.4)
No	6 (20.7)	4 (13.8)	12 (20.0)	22 (18.6)
History of alcohol use diagnosis				
Yes	17 (58.6)	24 (82.8)	44 (73.3)	85 (72.0)
No	12 (41.4)	5 (17.2)	16 (26.7)	33 (28.0)
AUDIT Total Score (*M, SD*, range) = 13.02, 4.70, 5–30 score				
Age (*M, SD*, range) = 21.14, 1.76, 18–25 years				

*Note.* “CIAN” stands for Call it a Night, the full digital sleep intervention, including personalized feedback and coaching, active diary self-monitoring, passive biosensor monitoring, and web-based advice. “A + SM” stands for advice plus self-monitoring control condition, which also includes passive monitoring. “A” stands for advice control condition, which also includes passive monitoring. “AUDIT” stands for Alcohol Use Disorders Identification Test^33^.

a There were no significant differences between trial groups on any demographic variable.

**Table 2:** CAN Thematic Analysis Salience (N = 107)

Thematic Category	Theme	*n* (%) ^a^
Helpful aspects of website	General helpfulness of website	69 (**64.5%** of 107)
	Helpful sleep-related information	69 (**64.5%** of 107)
	New information from website	58 (54.2% of 107)
	Reminders of known information	42 (39.3% of 107)
	Usefulness of website strategies	38 (35.5% of 107)
	Website understandability	28 (26.2% of 107)
	Helpful alcohol-related information	25 (23.4% of 107)
	User-friendliness of website	25 (23.4% of 107)
	Memorability of information	9 (8.4% of 107)
Unhelpful aspects of website	Lack of new information	14 (**13.1%** of 107)
	Irrelevance of information	5 (4.7% of 107)
	Difficulty implementing strategies	5 (4.7% of 107)
Helpful aspects of biosensors	Ankle biosensor increased awareness	36 (**33.6%** of 107)
	General helpfulness of biosensor(s)	35 (32.7% of 107)
	Watch biosensor increased awareness	33 (30.8% of 107)
Neutral aspect of biosensors	Neutral aspect of biosensors	65 (**60.7%** of 107)
Unhelpful aspects of biosensors	Burdensomeness of ankle biosensor	22 (**20.6%** of 107)
	Unhelpful without data feedback	16 (15.0% of 107)
	Lack of behavior change	12 (11.2% of 107)
	Burdensomeness of watch biosensor	8 (7.5% of 107)
	Stigma of ankle biosensor	6 (5.6% of 107)
	Ankle biosensor increased drinking	5 (4.7% of 107)
Helpful aspects of diaries	Increased mindfulness of sleep	42 (**53.8%** of 78)
	General helpfulness of diaries	38 (48.7% of 78)
	Increased mindfulness of alcohol use	31 (39.7% of 78)
	New experience of keeping a diary	22 (28.2% of 78)
	Ease of answering questions	17 (21.8% of 78)
	Motivation to change behaviors	10 (12.8% of 78)
Neutral aspect of diaries	Neutral aspect of diaries	13 (**16.7%** of 78)
Unhelpful aspects of diaries	Challenges answering questions	13 (**16.7%** of 78)
	Lack of behavior change	6 (7.7% of 78)
	Lack of new information	6 (7.7% of 78)
Helpful aspects of feedback	Helpfulness of personalization	45 (**90.0%** of 50)
	General helpfulness of feedback	29 (58.0% of 50)
	Helpful data presentations	27 (54.0% of 50)
Unhelpful aspects of feedback	Lack of new information	4 (**8.0%** of 50)
Most influential interventions	Feedback was most influential	36 (**72.0%** of 50)
	Website was most influential	39 (36.4% of 107)
	Diary was most influential	25 (32.1% of 78)
	Biosensors were most influential	17 (15.9% of 107)
	Multiple things were helpful	15 (14.0% of 107)
Suggestions for interventions	Willing to wear more devices (watch)	65 (**60.7%** of 107)
	Want diet and exercise-related feedback	63 (58.9% of 107)
	Willing to complete new diary questions	60 (56.1% of 107)
	Want caffeine-related feedback	53 (49.5% of 107)
	Want other potential feedback	33 (30.8% of 107)
	Want environment-related feedback	26 (24.3% of 107)
	Unwilling to add question and/or device	18 (16.8% of 107)
	Improvements for feedback	5 (10.0% of 50)
	Improvements for diaries	6 (7.7% of 78)
	Improvements for biosensors	8 (7.5% of 107)
	Improvements for website	4 (3.7% of 107)

*Note.* Theme salience (% of those who stated the theme) is based on the number of participants who answered the interview question that resulted in the theme. General questions and questions about the website and biosensors were asked of all 107 participants across trial groups. Questions about diaries were asked of the 78 *CIAN* and *A+SM* group participants who completed the diaries and the exit interviews. Questions about feedback were asked of the 50 *CIAN* participants who received feedback and completed the exit interview.

**Table 3:** CIAN Exit Survey Outcomes (N = 118)

Outcome (Agreement 1–5)	A	A+SM	CIAN	Total
	*M*(*SD*)	*M*(*SD*)	*M*(*SD*)	*M*(*SD*)
General program outcomes				
Overall satisfaction	4.45 (0.74)	4.48 (0.57)	4.60 (0.56)	4.53 (0.61)
Understandability	4.52 (0.87)	4.59 (0.83)	4.65 (0.66)	4.60 (0.75)
Schedule workability	4.66 (0.94)	4.62 (0.86)	4.67 (0.82)	4.65 (0.85)
Appropriate visit length	4.31 (1.11)	4.10 (1.14)	4.27 (0.92)	4.24 (1.02)
Lifestyle change promotion	4.03 (0.87)	4.10 (0.77)	4.23 (0.96)	4.15 (0.89)
Comfortability	4.76 (0.79)	4.66 (0.81)	4.57 (0.81)	4.64 (0.80)
Hope for change promotion	4.00 (0.89)	3.97 (0.91)	4.10 (1.02)	4.04 (0.96)
Importance of target habits	4.48 (0.87)	4.52 (0.69)	4.57 (0.81)	4.53 (0.79)
Overall effectiveness	4.00 (0.76)	4.48 (0.51)	4.55 (0.57)	4.40 (0.64)
Advice (A) outcomes				
Module 1 helpfulness	4.24 (0.74)	4.24 (0.64)	4.43 (0.65)	4.34 (0.67)
Module 2 helpfulness	4.07 (0.96)	4.31 (0.66)	4.45 (0.65)	4.32 (0.75)
User-friendliness	4.52 (0.51)	4.41 (0.50)	4.40 (0.94)	4.43 (0.76)
Comparability to other websites	4.76 (0.44)	4.52 (0.57)	4.73 (0.58)	4.68 (0.55)
Enjoyment	3.97 (0.91)	3.97 (0.73)	4.00 (0.77)	3.98 (0.79)
Passive-monitoring (biosensor) outcomes				
Actiwatch comfortable	3.93 (1.13)	4.14 (0.69)	3.92 (0.94)	3.97 (0.94)
Actiwatch not embarrassing	4.00 (1.16)	4.24 (0.64)	4.23 (0.79)	4.18 (0.86)
Actiwatch did not interfere with work	4.34 (0.97)	4.55 (0.51)	4.52 (0.62)	4.48 (0.70)
Actiwatch did not interfere with exercise	4.41 (0.78)	4.31 (0.66)	4.32 (0.91)	4.34 (0.82)
Actiwatch did not interfere with sleep	4.41 (0.68)	4.34 (0.67)	4.30 (0.89)	4.34 (0.79)
Actiwatch did not interfere with concentration	4.59 (0.57)	4.55 (0.57)	4.52 (0.60)	4.54 (0.58)
Actiwatch did not interfere with clothing	4.41 (0.87)	4.21 (0.98)	4.18 (1.02)	4.25 (0.97)
Actiwatch not noticeable	4.07 (1.00)	4.17 (0.93)	4.17 (0.96)	4.14 (0.95)
Actiwatch did not change routine	4.59 (0.57)	4.38 (0.82)	4.28 (1.01)	4.38 (0.88)
Actiwatch wear more	4.24 (0.87)	4.34 (0.81)	4.27 (0.92)	4.28 (0.88)
Ankle monitor comfortable	2.15 (0.95)	2.66 (1.11)	2.60 (1.08)	2.51 (1.07)
Ankle monitor not embarrassing	2.59 (1.19)	2.66 (1.42)	2.98 (1.38)	2.81 (1.35)
Ankle monitor did not interfere with work	3.37 (1.08)	3.79 (1.05)	4.05 (1.00)	3.83 (1.06)
Ankle monitor did not interfere with exercise	2.48 (1.16)	3.03 (1.30)	3.02 (1.32)	2.90 (1.29)
Ankle monitor did not interfere with sleep	3.04 (1.19)	3.34 (1.14)	3.25 (1.16)	3.22 (1.16)
Ankle mon. did not Interfere with concentration	3.56 (1.25)	3.97 (0.91)	4.05 (1.02)	3.91 (1.06)
Ankle monitor did not interfere with clothing	2.33 (1.47)	2.17 (1.28)	2.55 (1.43)	2.41 (1.40)
Ankle monitor not noticeable	2.85 (1.22)	2.66 (1.37)	2.60 (1.24)	2.67 (1.26)
Ankle monitor did not change routine	3.11 (1.25)	3.21 (1.18)	3.10 (1.36)	3.12 (1.28)
Ankle monitor wear more	3.26 (1.53)	2.93 (1.25)	3.42 (1.37)	3.26 (1.38)
Self-monitoring (SM) outcomes				
Diary helpfulness	-	4.07 (0.72)	4.19 (0.79)	4.15 (0.76)
Diary easiness	-	4.34 (0.61)	4.58 (0.56)	4.51 (0.59)
Diary not burdensome	-	3.97 (1.09)	4.17 (0.91)	4.10 (0.97)
Diary did not change schedule	-	4.24 (0.83)	4.27 (1.06)	4.26 (0.98)
Diary easy to remember	-	3.72 (1.16)	3.92 (1.00)	3.85 (1.05)
Diary enjoyable	-	3.52 (0.91)	3.83 (0.91)	3.73 (0.91)
Diary would continue	-	3.52 (1.15)	3.83 (0.92)	3.73 (1.01)
Feedback (F) outcomes				
Sleep feedback helpfulness	-	-	4.70 (0.53)	4.70 (0.53)
Alcohol feedback helpfulness	-	-	4.65 (0.55)	4.65 (0.55)

*Note.* “CIAN” stands for Call it a Night, the full digital sleep intervention, including personalized feedback and coaching, active diary self-monitoring, passive biosensor monitoring, and web-based advice. “A + SM” stands for advice plus self-monitoring control condition, which also includes passive monitoring. “A” stands for advice control condition, which also includes passive monitoring.

Total means and standard deviations are based on 118 participants who completed the exit interview, including 60 CIAN, 29 A + SM, and 29 A participants. Some items had minor levels of missingness (< 5 participants).

## Data Availability

The datasets used and analyzed during the current study are available from the corresponding author on reasonable request.
